# Cannabis use disorder: from neurobiology to treatment

**DOI:** 10.1172/JCI172887

**Published:** 2024-10-15

**Authors:** Bernard Le Foll, Victor M. Tang, Sergio Rueda, Leanne V. Trick, Isabelle Boileau

**Affiliations:** 1 Institute for Mental Health Policy Research, Centre for Addiction and Mental Health, Toronto, Ontario, Canada.; 2 Department of Pharmacology and Toxicology, University of Toronto, Toronto, Ontario, Canada.; 3 Translational Addiction Research Laboratory, Centre for Addiction and Mental Health, Toronto, Ontario, Canada.; 4 Campbell Family Mental Health Research Institute Centre for Addiction and Mental Health, Toronto, Ontario, Canada.; 5 Institute of Medical Sciences,; 6 Department of Psychiatry, and; 7 Department of Family and Community Medicine, University of Toronto, Toronto, Ontario, Canada.; 8 Department of Psychology, Durham University, Durham, United Kingdom.; 9 Brain Health Imaging Centre, Toronto, Ontario, Canada.

## Abstract

Cannabis has been legalized for medical and recreational purposes in multiple countries. A large number of people are using cannabis and some will develop cannabis use disorder (CUD). There is a growing recognition that CUD requires specific interventions. This Review will cover this topic from a variety of perspectives, with a particular emphasis on neurobiological findings and innovative treatment approaches that are being pursued. We will first describe the epidemiology and burden of disease of CUD, including risk factors associated with CUD (both in terms of general risk and genetic risk variants). Neurobiological alterations identified in brain imaging studies will be presented. Several psychosocial interventions that are useful for the management of CUD, including motivational enhancement therapy, behavioral and cognitive therapy, and contingency management, will be covered. Although no pharmacological interventions are yet approved for CUD, we present the most promising pharmacological interventions being tested.

## Epidemiology of cannabis use disorder

Cannabis ranks among the most used psychoactive substances globally, following only caffeine, alcohol, and tobacco (1). An estimated 219 million people between the ages of 15 and 64 years worldwide used cannabis in 2021, representing 4.3% of the global population in that age range (2). That same year, in the United States, a large, nationally representative survey estimated that 52.4 million people aged 12 years or older (18.7% of individuals in that age range) used cannabis in the past year (3). Furthermore, 16.3 million people (5.8% of individuals aged 12 years or older) met criteria for cannabis use disorder (CUD) (3), a chronic and relapsing condition characterized by persistent cannabis use despite adverse consequences (4). Although CUD is present across all age groups, it predominantly affects young adults. The proportion of young adults aged 18–25 years with past-year CUD (14.4%) was higher than the proportion of adolescents aged 12–17 years (4.8%) or adults 26 years or older (4.6%) (3). The median age of onset for CUD was 22 years (interquartile range [IQR], 19–29 years) (5). A younger age of initiation of cannabis use is associated with a faster progression to CUD, potentially leading to a more severe manifestation of the disorder (6, 7). Across all age groups, male individuals are also more likely to develop CUD (8–10), but some preliminary evidence suggests that female individuals progress to CUD more rapidly after initiation (8, 11).

The diagnostic criteria for CUD have changed over time. The DSM-5 Substance-Related Disorders Work Group changed the structure of CUD from two disorders as defined in the DSM-IV (i.e., abuse and dependence) to a single disorder that combined 11 criteria, adding craving and withdrawal and removing substance-related legal problems (12). The latest edition, published in 2022 (DSM-5-TR), includes items related to impaired control over cannabis use, social impairments due to cannabis use, risky use of cannabis, and pharmacological indicators (13). Based on the number of criteria, CUD is now graded as mild (1–3 criteria), moderate (4-5 criteria), and severe (6 criteria). Because reports from national surveys and other large-scale population health studies still report findings based on previous DSM versions, it is important to keep in mind that these constructs include similar content but are organized differently.

The risk of developing CUD is influenced by various factors (1). A recent meta-analysis of observational studies with general population samples showed that people who have consumed cannabis (lifetime, recent, or regular use) have a 1 in 5 risk of developing CUD (14). The pooled prevalence estimate for CUD was 22% (95% CI: 18%–26%), and the risks were higher for younger people and for those who used cannabis daily or weekly. Modifiable factors influencing the onset of CUD include the frequency and duration of cannabis use. A recent meta-analysis pooling data from six prospective longitudinal studies found a log-linear dose-response relationship between four categorical levels of frequency of use (yearly, monthly, weekly, and daily) and the development of CUD (15). The risk of CUD increased 8-fold from a relative risk [RR] of 2.03 (95% CI, 1.85–2.22) for yearly use to a RR of 16.99 (95% CI, 11.80–24.46) for daily use. Multilevel modeling showed an absolute risk increase (ARI) from 3.5% (95% CI, 2.6–4.7) for past-year use to 36% (95% CI, 27.0–47.9) for daily use, suggesting that one-third of daily cannabis users are expected to develop CUD (15). This study showed not only that relatively infrequent use can result in CUD, but that the risk significantly increases with every additional level of use.

A comprehensive exploration of other plausible modifiable risk factors, such as amounts used or potency of Δ^9-^tetrahydrocannabinol (THC), the principal psychoactive component of cannabis, are difficult to carry out due to challenges in assessing the quantity and potency of THC content in cannabis products. Cannabis availability and use has been shifting from flower to processed products and from lower to higher THC products. Jurisdictions that have legalized cannabis have imposed minimal constraints on product availability such that there are no limits on THC concentrations (with the exception of edibles) (16). There have been recent increases in the use and availability of concentrates, with THC levels averaging 65%–70% but reaching levels as high as 90%–95% (17–20). Conversely, in cannabis flowers, THC concentration typically ranges between 16% and 21% but can go as high as 25%–30% (18, 21), and new lines of concentrate-infused flower products can go as high as 50%–55% (22). Despite the wider availability of high-THC products (23), there is limited experimental evidence on their effects (24). However, a recent systematic review reported low-quality evidence, suggesting that higher potency cannabis use was associated with an increased risk of CUD (25).

CUD frequently co-occurs with other psychiatric conditions, including various substance use disorders. Approximately three-quarters (73.8%) of patients diagnosed with CUD in primary care concurrently experience at least one other substance use disorder, predominantly those involving alcohol or tobacco (26). A recent systematic review focusing on large US population–based surveys reported large adjusted odds ratios (aORs) representing strong associations between past-year CUD and other substance use disorders, including any other substance use disorder (aOR = 9.3, 95% CI, 7.70–11.21), alcohol use disorder (aOR = 6.0, 95% CI, 5.10–6.97), and nicotine use disorder (aOR = 6.2, 95% CI, 5.24–7.34) (27). In addition, people diagnosed with CUD often exhibit other concurrent psychiatric disorders. The same review of large epidemiological studies also reported strong associations between past-year CUD and major depressive disorder (aOR = 2.8; 95% CI, 2.33–3.41), bipolar I disorder (aOR = 5.0; 95% CI, 3.65–6.75), any anxiety disorder (aOR = 2.8; 95% CI, 2.24–3.39), panic disorder (aOR = 3.3; 95% CI, 2.50–4.48), generalized anxiety disorder (aOR = 3.7; 95% CI, 2.79–5.02), posttraumatic stress disorder (aOR = 4.3; 95% CI, 3.26–5.64), and any personality disorder (aOR = 4.8; 95% CI, 3.96–5.75) (27). As epidemiological studies cannot address causality, Mendelian randomization studies enable inference of causality between cannabis use and subsequent risk of psychiatric disorders (28). However, recent large studies have reported weak evidence for causal effect of cannabis use leading to increased risk of schizophrenia, while finding a more robust causal effect in the other direction (29, 30) (but see ref. 28). Other Mendelian randomization studies could not detect significant increase of risk of depression (31) or of bipolar disorder (32) induced by cannabis use. The presence of a co-occurring psychiatric disorder is linked to heightened severity of CUD and diminished responsiveness to treatment.

## Burden of disease

Cannabis use contributes to a global health burden, although notably less than other psychoactive substances such as alcohol, tobacco, opioids, and stimulants. According to the Global Burden of Disease project, in 2016 CUDs resulted in an approximate 646,500 years of life lost to disability, with an age-standardized rate of 8.5 years per 100,000 persons (33). Despite an increase in cardiovascular disease mortality among US adults (34), the association between cannabis use and increased all-cause mortality remains uncertain (35). In Canada, the cannabis-attributable burden of disease in 2012 included 55,813 years of life lost due to disability, 10,533 years of life lost due to premature mortality, and 66,346 disability-adjusted life years overall. CUD was the most important single cause of cannabis-attributable burden of disease and the largest contributor to morbidity and years of life lost due to disability while cannabis-attributable lung cancer was the largest contributor to mortality followed by motor vehicle accidents (36). Guidelines have been developed to reduce the effect of cannabis on populations. Those cannabis use guidelines aimed at lowering risk have been endorsed by some public agencies and widely disseminated in Canada to reduce the impact of cannabis legalization using a public health framework (see Table 1) (37). It should be noted that high potency cannabis products may require specific measures to limit their effect on population (38).

## Pharmacology

**Figure 1:**
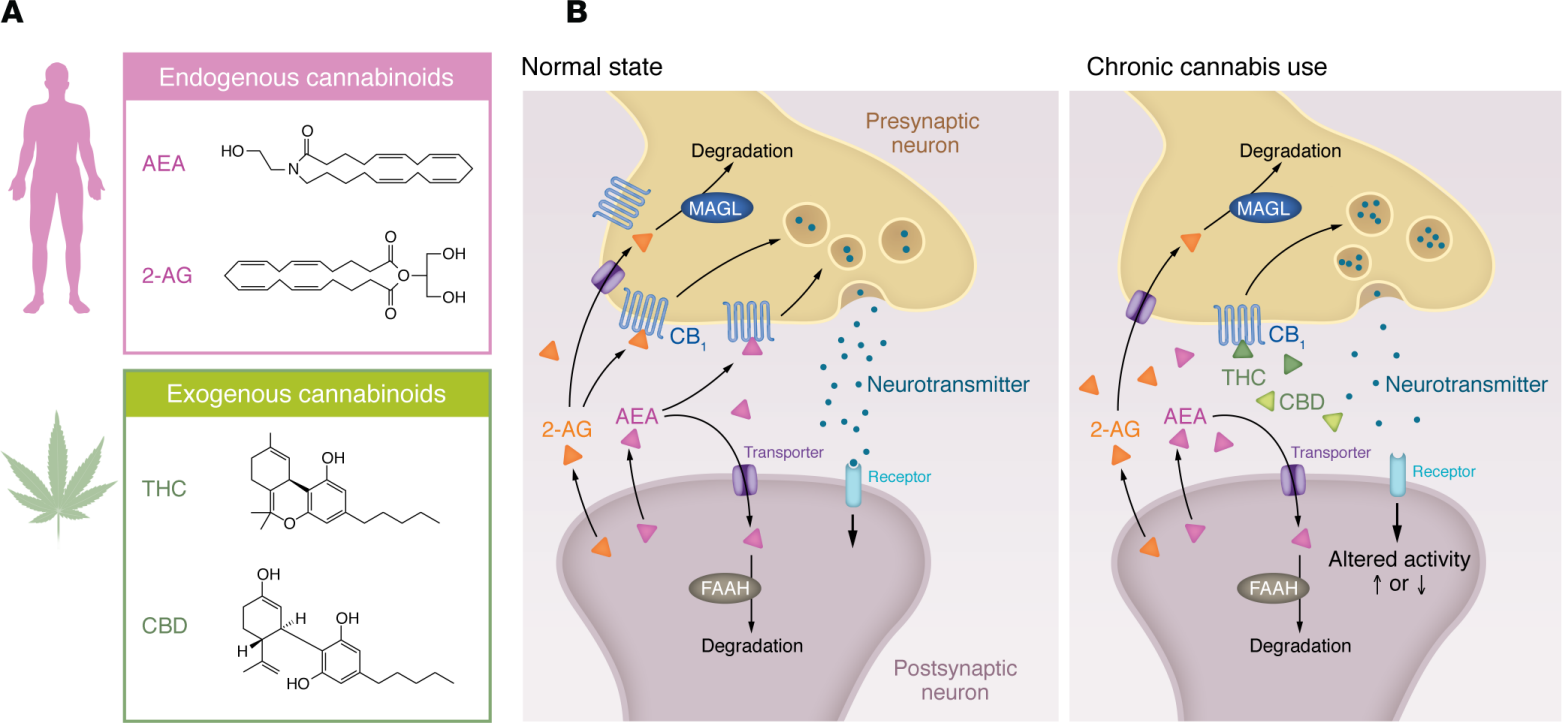
Signaling by endogenous and exogenous cannabinoids modifies synaptic activity at multiple levels. (**A**) There are two known endocannabinoids, called anandamide (AEA) and 2-arachidonoylglycerol (2-AG). Cannabis contains exogenous cannabinoids, including Δ^9^-tetrahydrocannabinol (THC) and cannabidiol (CBD). (**B**) Endogenous cannabinoid release prevents overstimulation of neurons, modulates the release of various neurotransmitters such as GABA and glutamate, and has downstream effects, notably on dopaminergic transmission. The enzyme fatty acid amide hydrolase (FAAH) degrades anandamide. The enzyme MAGL regulates 2-AG. THC stimulates the cannabinoid system by binding to CB_1_ and CB_2_ receptors. Compared with signaling by endogenous cannabinoids (normal state), chronic cannabis use likely results in changes in various components of the endocannabinoid system (e.g., CB_1_ and FAAH).

The endocannabinoid system is present in the brain and periphery. THC primarily exerts its effects by acting as a partial agonist at the widely expressed Gi/Go protein–coupled cannabinoid receptor subtype 1 (CB_1_) (39), but it is also a partial agonist for CB_2_. The psychomimetic effects of THC are mediated by CB_1_. Two endocannabinoid neurotransmitters have been identified: *N*-arachidonoylethanolamine (AEA or anandamide) and 2-arachidonoylglycerol (2-AG). There are enzymes that regulate the synthesis and the degradation of those endocannabinoids (e.g., fatty acid amide hydrolase [FAAH] degrades anandamide; see Figure 1). Certain characteristics of CUD are thought to emerge, in part, due to molecular adaptations in the brain resulting from repetitive exposure to cannabis, particularly its primary psychoactive compound, THC. One of the most noteworthy and consistently observed findings of adaptations to chronic cannabis use is the desensitization of CB_1_ receptors in preclinical models (40). CB_1_ desensitization refers to a reduced responsiveness or sensitivity of CB_1_ receptors to the binding of cannabinoids over time. In preclinical models, this phenomenon has been associated with the development of significant tolerance to cannabis and to the severity of withdrawal symptoms. As well, the reduction in CB_1_ signaling is believed to impact various physiological and behavioral processes, including appetite, memory and learning, mood, pain perception, and sleep (40). CB_1_ desensitization in preclinical models may also alter neurotransmitter release patterns and synaptic communication (40).

## Preclinical addiction models

Most drugs that have addictive potential are self-administered by laboratory animals (rodents or nonhuman primates) in experimental settings. However, initial attempts to develop a model of THC self-administration in rodents have been unsuccessful (41). The first clear intravenously THC self-administration has been obtained in squirrel monkeys previously trained to self-administer psychostimulant drug, but it has been also shown in naive animals (42, 43). Other preclinical models (e.g., conditioned place preference, drug discrimination, withdrawal paradigms, or intravenous self-administration of the direct CB_1_ agonist WIN55,212-2) have been used to study the neurobiological mechanisms underlying CUD (see ref. 41 for a review). It is likely that the recent findings that vaporized cannabis extracts have reinforcing properties and are able to generate conditioned drug-seeking in rats will lead to further discoveries, as this will provide a useful and maybe more valid model to study relapse phenomenon (44).

**Figure 2:**
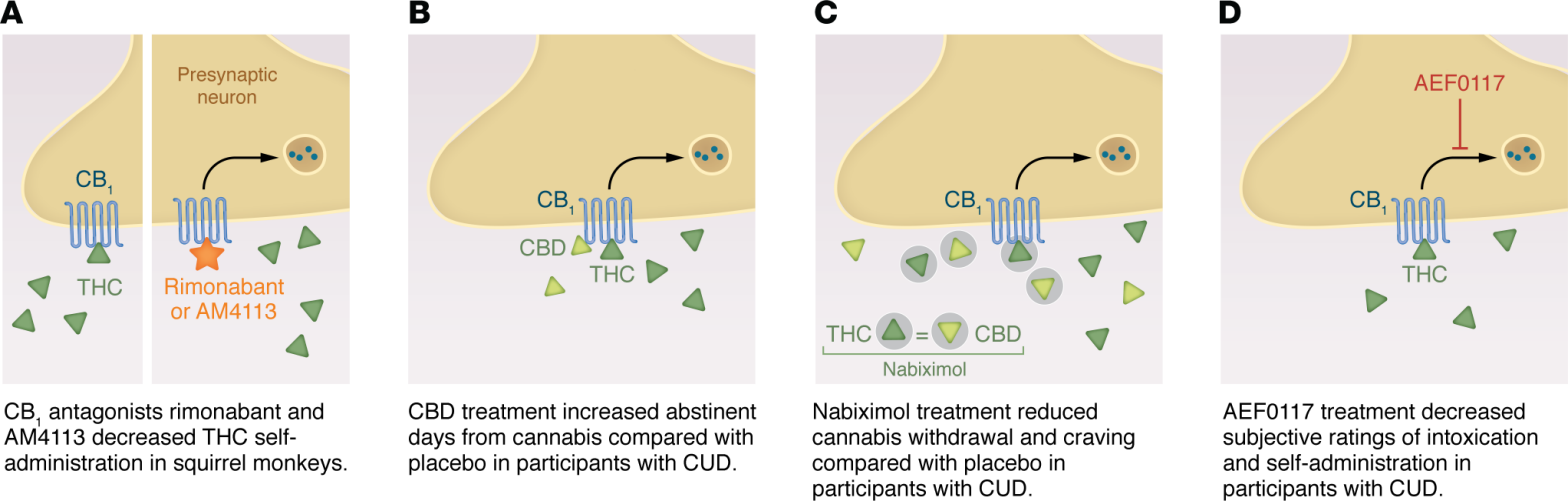
Selected pharmacological strategies under investigation for treatment of CUD. (**A**) CB_1_ antagonists have demonstrated efficacy in preclinical models, with variable tolerability profiles. (**B**) CBD is the main nonpsychomimetic cannabis-derived compound. It has shown promising results in treatment of CUD. (**C**) Nabiximol (a 1:1 mixture of THC and CBD) may facilitate abstinence from cannabis in treatment-seeking patients, possibly by reducing withdrawal. (**D**) AEF0117, which blocks the intracellular signaling of CB_1_, showed promise in decreasing cannabis use in a phase IIA study of volunteers with CUD.

The preclinical models that are used to study neurobiological mechanisms underlying CUD are also used to screen the utility of possible medications that can be tested in humans. Those studies notably point to a critical role of CB_1_. Blockade of the CB_1_ by the inverse agonist rimonabant prevented the elevation of dopamine induced by THC (45) but also THC taking (46, 47) and THC seeking (47) (see Figure 2A). However, chronic administration of rimonabant led to adverse psychiatric events, which resulted in its withdrawal from the market. Rimonabant was therefore used only for a few years in Europe and never marketed in North America (48). At the present time, various investigators are pursuing other ways of modulating the CB_1_ transmission that may be devoid of the psychiatric side-effects of rimonabant. For example, AM4113 is a neutral CB_1_ antagonist has been developed that is able to reduce THC taking and THC seeking in squirrel monkeys (47) (Figure 2A), but it appears to have a better tolerability profile (49). Pregnenolone is a drug that can block some effects of THC by acting as a signaling-specific inhibitor of CB_1_ (CB_1_-SSi) (50). A pregnenolone derivative drug called AEF0117, a more promising CB_1_-SSi (51), reduces THC taking and THC seeking as well as THC-induced elevation of dopamine and various measures of impairment induced by THC (51). Negative allosteric modulators (NAMs) for CB_1_ may have some therapeutic utility by blocking some effects of THC (but not all; for instance, there was no induction of withdrawal) (52). Cannabidiol is the major nonpsychomimetic compound derived from cannabis that has some potential for a range of neuropsychiatric disorders, including addictive disorders (53). However, the preclinical findings for CUD are mixed (45, 54–56).

Another approach consists of stimulating CB_1_ transmission. This could be achieved by administration of CB_1_ agonists such as THC or other derivatives (THC has been shown to be able to reverse pharmacologically induced cannabinoid withdrawal, ref. 57) or by modulating (57) the endocannabinoid tone, e.g., by blocking degradation processes. Blocking FAAH enzyme would enhance anandamide levels, while blocking MAGL enzyme would enhance 2-G levels. However, it appears that the two main endocannabinoids (2-AG and anandamide) may have opposite effects on their control of dopamine activity and reward seeking (58) and may modulate drug seeking differently (58–60). It is unclear how those two approaches would modulate THC taking, and THC seeking, and withdrawal at this point.

Other preclinical studies have identified various potential alternative approaches. Blocking mu opioid receptor signaling reduces elevation of dopamine induced by THC (45) and THC self-administration (61). Enhancing endogenous brain levels of kynurenic acid has a similar promising profile (62). It is likely that more targets will be identified as preclinical models become more widely used and with enhanced interest on this topic.

## Heritability and genetic factors

The heritability of CUD has been recognized in early family-linkage and twin studies, with genetic factors accounting for 40%–70% of the risk of the disorder (63, 64). Genetic contributions have also been identified for cannabis use and cannabis use initiation (63), although genetic liability to CUD appears to only partially overlap with genetic correlates of cannabis use (65). Other phenotypes (e.g., subjective effects) may also be affected by gene variants (66, 67). GWAS approaches to identifying genes implicated in CUD initially did not show any single-nucleotide polymorphisms (SNPs) to be genome-wide significant (68), until an analysis of 14,754 patients identified significant SNP associations in 3 regions, which included an antisense transcript (rs143244591), and genes involved in calcium signaling (rs146091982) and growth cones during CNS development (rs77378271) (69). A subsequent study found a different cluster of associated SNPs on chromosome 10 in a cohort of individuals of European ancestry, and this was replicated in an independent cohort of African but not in European individuals (70). Demontis and colleagues (71) presented compelling findings in a GWAS that implicated a risk locus for CUD on chromosome 8 for the cholinergic receptor nicotinic α2 subunit gene (*CHRNA2*) that was then replicated in an independent sample. A recent larger GWAS further confirmed this finding with *CHRNA2* and also found a risk locus on chromosome 7 with *FOXP2* (65), which encodes a protein essential for synaptic plasticity and has been associated with externalizing behaviors and risk-taking behaviors (72). Furthermore, a finding consistent across studies is the shared genetic liability of CUD with other psychiatric illnesses such as major depressive disorder and schizophrenia (65, 69, 73). Most recently, a larger GWAS study identified some promising genes involved in CUD risk but also noted that this finding may be influenced by ancestry (see Table 2) (73). Overall, these findings indicate that CUD is likely a polygenic disorder, and more research is needed in this area.

## Neurophysiology

On a functional neurophysiological level, it is possible to measure the functioning of the brain by recording electrical activity using electroencephalography (EEG). Individuals with CUD have abnormal inhibitory control as measured by task-based EEG measures of frontal α asymmetry (74, 75). Furthermore, attentional biases toward cannabis-related cues demonstrated frontal EEG changes that were greater than that induced by both negative and neutral stimuli (76). Changes on frontal EEG related to cognitive deficits, such as reduced selective attention and processing speed, show that increasing frequency and chronicity of use is associated with greater abnormalities (77, 78). Interestingly, it has been shown that positivity on frontocentral electrode sites following reward receipt was increased in occasional cannabis users but not in individuals with CUD (79), suggesting that the progression from cannabis use to CUD may reflect a gradual hypoactivation to reward. On a network level, EEG has also been used to understand the differences in functional connectivity in the brains of individuals with CUD. Analyses of synchronization between distributed signals in the salience network and the central executive network revealed correlation with the degree of problematic cannabis use (80). In another study of spontaneous EEG activity at rest, CUD was shown to be associated with greater EEG complexity across brain regions, which reflects greater disorganization and noncoherent activity. Importantly, this finding was evident only in cannabis dependence and not in cannabis users who were not dependent (81).

The functioning of the brain has been also studied using transcranial magnetic stimulation. This approach uses electromagnetic pulses to depolarize focal areas of the cerebral cortex. Repetitive transcranial magnetic stimulation administration leads to a change in observed corticospinal excitability, which is a normal adaptive function of the brain. Cannabis use and CUD have been associated with a reduced capacity for cortical inhibition, a response known to involve γ-aminobutyric acid (GABA) receptors (74–78, 82–84).

## fMRI studies

Various brain imaging studies have been conducted in regular cannabis users. A recent systematic review of fMRI studies looking at cue reactivity identified 18 studies (comprising 603 cannabis users and 315 individuals acting as controls) (85). Those studies indicated that exposure to cannabis-related stimuli versus neutral stimuli produces greater brain activation of three principal brain areas: the striatum, the prefrontal cortex, and the parietal cortex (85). Other areas such as hippocampus, amygdala, thalamus, and occipital cortex are also involved (85). These findings are consistent with the pattern of brain activations induced by drug-associated cues in individuals with other addictions and support the notion that addiction processes recruit a vast number of brain areas to mediate cravings and drug-seeking behaviors (1, 86). The few studies that have explored the effect of chronic cannabis use on brain volumes (87) showed marginal effects that may be sex dependent (87) and much more limited than the impact of alcohol (88). Studies with larger samples are required.

## PET studies

PET can be used to investigate the endocannabinoid system in the living human brain. PET probes have been developed that allow quantification of the CB_1_ receptor and more recently the enzyme FAAH, which degrades the endogenous cannabinoid anandamide (1, 89). Some studies have investigated the regulation of those targets in individuals with CUD. Three published studies have investigated CB_1_ receptor status in people with CUD (*n* = ~50 individuals with chronic cannabis use) using three different radiotracers: [^11^C]OMAR, [^18^F]MK-9470, and [^11^F]FMPEP-d 2 (for a review of these tracers see, ref. 89). The findings are in line with preclinical data and suggest that chronic cannabis use is linked with lower CB_1_ tracer binding (90–92). However, the studies are inconsistent regarding regional specificity of these effects. One study noted reduced CB_1_ binding in cortical regions (91), another identifies lower binding affecting the hippocampus, amygdala, cingulate, and insula (92), while a third study showed a more global effect (90). A recovery in CB_1_ receptor binding is observed following a four-week period of monitored cannabis abstinence (91). In these studies, the downregulation of CB_1_ receptors was not conclusively linked to withdrawal symptoms. Instead, it was associated with the duration of cannabis use (91) and with increased anger and hostility in female cannabis users (92).

Limited preclinical investigations have explored the impact of chronic exposure to THC and subsequent withdrawal on the activity of FAAH, the enzyme responsible for metabolizing the major endocannabinoid anandamide. Most of these studies suggested that subchronic exposure to THC is associated with decreased FAAH activity (with a few exceptions) and elevated anandamide levels, particularly in the limbic forebrain but not in the striatum (93–95). The exact mechanism triggering this reduction in FAAH activity remains unclear and may involve a homeostatic response to CB_1_ desensitization. Lowering FAAH activity can influence cannabis withdrawal (96), and understanding its status in the living human brain is crucial. Only two studies have investigated FAAH levels in individuals with chronic cannabis use (97, 98). Collectively testing around 23 individuals with CUD, these studies have demonstrated widespread reductions in FAAH binding among cannabis users, a phenomenon linked to the severity and chronicity of cannabis use.

Molecular imaging studies have also examined the dopaminergic system in individuals with CUD, given its pivotal role in reward processing and its implicated involvement in the development of addiction. PET imaging studies in the area of addiction have consistently reported dopamine system impairments, particularly in cases of psychostimulant use disorder (99). In CUD, PET studies of the dopamine system have utilized tracers targeting D_2/3_ receptors, such as [^11^C]raclopride and [^11^C]-(+)-PHNO, along with [^18^F]DOPA, a tracer that reflects dopamine synthesis. One study’s cumulative findings suggested a decrease in dopamine synthesis among cannabis users, a phenomenon linked to more intensive cannabis use (99). Furthermore, two studies (100, 101) observed diminished stimulant-induced dopamine release in CUD, which contrasts with a third study that did not (102). Interestingly, in multiple studies, D_2/3_ receptor status did not appear to be lower in CUD, according to studies by Sevy et al. (2008) (103), Volkow et al. 2014 (101), Tomasi et al. (2015) (104), and Urban, et al. (2012) (102).

Our understanding of the molecular underpinnings of CUD in living humans is currently restricted, especially regarding the connection between variability in the endocannabinoid system or dopamine markers and the manifestation of CUD symptoms and phenotypes. Further research endeavors utilizing novel molecular imaging techniques and comprehensive clinical assessments are needed to bridge these knowledge gaps.

## Psychological interventions

In the absence of an approved pharmacotherapy for CUD, psychological and psychosocial interventions are currently the primary treatment options. These include motivational, cognitive, and behavioral approaches that were originally developed for the treatment of other substance use disorders and other individual, community, or family interventions (e.g., drug counseling, peer support, family therapy).

Previous meta-analytic reviews, including a Cochrane review (105–108), have pooled the findings of randomized controlled trials investigating the effectiveness of psychological treatments for CUD compared with active and inactive control conditions among treatment-seeking and nontreatment-seeking adults and young people. These reviews highlight the relatively small size of the evidence base (the number of relevant individual trials identified ranged from 5 to 23) but demonstrate that, overall, psychological interventions lead to modest reductions in the frequency and quantity of cannabis use (although evidence for improvements in other cannabis-related outcomes is less consistent). Psychological interventions identified in the studies included in these reviews include motivational enhancement therapy (MET), cognitive behavioral therapy (CBT), relapse prevention (a cognitive behavioral approach focused on prevention and management of lapses in abstinence), contingency management (CM), social support, mindfulness-based meditation, drug education and counseling, and various combinations of these interventions.

MET and CBT are the most widely researched individual psychological treatments for CUD. The aim of MET is to enhance motivation to stop or reduce cannabis use and increase self-efficacy through a combination of psychoeducation, goal setting, and developing plans for change, delivered within an empathic and nonjudgmental environment. CBT focuses on identifying both external triggers for cannabis use and unconstructive patterns of thought and behavior that maintain cannabis use and encourages the development of adaptive cognitive, behavioral, and emotional skills (such as coping strategies, problem-solving, and emotion regulation). Trials have shown that individually both MET and CBT lead to modest improvements in cannabis-related outcomes (including reduced frequency and quantity of cannabis use, a higher proportion of days of abstinence, fewer symptoms of dependence, fewer cannabis-related problems, and increased confidence to change cannabis use) among treatment seekers and nontreatment seekers (109–114), including individuals with psychiatric comorbidity (115). However, interventions that combine elements of both MET (to facilitate initial abstinence) and CBT (to support continued abstinence) are increasingly being recommended (116–119). Such suggestions are empirically supported, for example, by the large multisite trials among cannabis smokers with CUD reported by Babor and colleagues (112) and Hoch et al. (120, 121). These trials showed that MET and CBT, combined with case management and problem-solving, respectively, improved outcomes that include the frequency of cannabis use, abstinence rates, and symptom severity compared with a wait list control condition, with treatment benefits observed at follow-up appointments 3 to 15 months after initiation of treatment (although effect sizes decreased as the length of follow-up increased in both studies). In addition, a recent observational study showed that following a 12-week MET and CBT intervention delivered specifically in a real-life group treatment setting, the quantity and frequency of cannabis use were both reduced and other cannabis-related outcomes (e.g., cannabis-related problems, craving, anxiety and depressive symptoms) improved compared with those before treatment (122). Combined motivational and CBT-based approaches have also been shown to reduce the quantity and frequency of cannabis use when delivered remotely (123, 124).

The utility of CM for treating CUD has been explored. CM is a behavioral intervention that utilizes financial or other incentives to positively reinforce abstinence, or other desirable target behaviors such as treatment attendance, and has yielded beneficial effects in other substance use disorders with during treatment (although these appear to wane as time since treatment increases) (125). Currently, few studies have investigated the effectiveness of CM for CUD specifically. Trials that included a CM-only condition showed a reduction in self-reported and objectively measured abstinence in comparison to other active treatment and control conditions among individuals with CUD (109, 126, 127). A recent, small observational study suggested that remote delivery of a CM intervention may be a feasible and effective treatment approach (128). Several studies have used CM in combination with other active treatments (such as CBT and MET) to investigate possible cumulative treatment gains. Overall, most of these studies indicate that combination treatments that include CM are superior to individual interventions in individuals with CUD (127, 129), including among young adults involved in the criminal justice system (130) and adults with psychiatric comorbidities (131). However, consistent with findings in other substance use disorders (125), the improvements observed during individual or adjunct CM treatment tend to diminish relatively rapidly after the cessation of treatment. Thus, it is unclear to what extent CM promotes long-term abstinence from cannabis use.

While previous studies demonstrate that psychological interventions for treating CUD have positive effects on cannabis-related outcomes, methodological weaknesses that have been highlighted limit the generalizability of the findings. These include high rates of dropout from treatment, heterogeneity in outcomes, and concerns about blinding of treatment allocation and outcome assessment (108). There are several other limitations of the existing evidence base. Chief among these are that effect sizes are often moderate at best (and tend to be highest where psychological treatments are compared against wait list or inactive control conditions, refs. 106, 108) and continuous abstinence rates are typically low, suggesting that although treatments are helpful in reducing cannabis use, they are not optimal for promoting complete abstinence. Additionally, the majority of existing studies have not included follow-up assessments beyond 12 months from treatment, and outcomes have tended to be most positive at the end of treatment or at short-term follow-up (e.g., refs. 112, 121). The effects of treatment over the long-term therefore require further investigation and it appears that sustained abstinence remains problematic. The addition of “booster” sessions after treatment may extend positive treatment effects. For example, following 9 sessions of MET and CBT, improved abstinence rates and fewer days of cannabis use were observed among adults with CUD who received maintenance checks at 1 and 4 months after treatment, compared with those in a “no-check” control condition (132).

The optimal duration and intensity of treatments also remains to be confirmed. Previous studies have delivered interventions of up to 14 sessions, although typically studies involving brief interventions (usually 1 or 2 sessions) have demonstrated the most inconsistent or null effects compared with inactive control conditions (133–135) and poorer outcomes compared with longer duration treatments (112). Further, poor rates of treatment retention in many studies (estimates suggest that as many as one-third of patients with substance use disorders including CUD drop out from psychosocial treatments, ref. 107) make it difficult to draw reliable conclusions about the number of sessions required to improve cannabis use outcomes.

To date, mechanisms of therapeutic change (136) and predictors of treatment outcomes have received scant attention in trials of psychological interventions for CUD. Preliminary findings from a recent observational study suggested that client factors including heavier cannabis use and elevated anxiety at entry to treatment may be linked to poorer treatment retention and greater posttreatment cannabis use (122). In addition, there were greater reductions in cannabis use in a trial of integrated MET and transdiagnostic CBT for both CUD and anxiety symptoms among dually diagnosed individuals, compared with standard MET and CBT, specifically among the subgroup with the most severe cannabis use at baseline (137). Better characterization of the factors that influence treatment effectiveness and engagement will be important in future studies as they could help to personalize and optimize treatments.

In summary, despite a relatively small evidence base, psychological interventions for CUD appear to be moderately effective, and combination treatments that both strengthen initial resolve to quit and support continued abstinence appear to be particularly helpful. However, helping individuals with CUD to achieve sustained abstinence remains problematic, and features of the intervention and characteristics of the population that are important for predicting treatment success remain poorly understood.

## Pharmacological interventions

In comparison to other drugs of abuse, many fewer clinical trials have been conducted to test the utility of pharmacotherapies for CUD. However, this area is currently expanding (see ref. 138 for a review). Two Cochrane reviews have been performed summarizing the evidence (139, 140). It should be noted that no pharmacological interventions have been approved yet for treatment of CUD. Although the number of trials is limited, it appears that antidepressants, anxiolytics, or mood stabilizers have no or limited utility in managing CUD. The most promising agents appear to target the cannabinoid system. Notably, CB_1_ agonists appear to be effective in attenuating the severity of cannabis withdrawal. This appears to be the case for direct CB_1_ agonists such as dronabinol or nabilone (141, 142), for nabiximol (a combination of THC with cannabidiol) (143), and for FAAH inhibition (96). Managing cannabis withdrawal with pharmacological tools may be useful at the beginning of treatment in some patients with severe CUD for which the intensity of withdrawal may prevent behavioral change (see ref. 144 for a review). However, the long-term utility of direct cannabinoid agonists such as dronabinol is unclear (142). Recent promising studies suggest that nabiximol may be helpful in treatment-seeking patients trying to abstain from cannabis (145–148) (Figure 2B).

PF-04457845, a FAAH inhibitor, has been tested in a single-site study with promising results (96). Following those findings, a multicenter trial was performed, recruiting 116 individuals in the active group and 112 individuals in the placebo group (NCT03386487). Participants were randomized to either placebo or to 4 mg PF-04457845 for eight weeks. Although the results are not yet published, some findings have been posted on Clinicaltrials.gov, and it appears that the primary outcome was negative (no apparent change in the average number of times per day of self-reported cannabis consumption based on the time-line follow back).

Cannabidiol has also been tested alone for CUD and appeared to be superior to placebo in a phase II study (149) (Figure 2C).

To our knowledge, neutral antagonists (such as AM4113) or NAMs have not yet been tested in humans. Recently, the CB_1_-SSi AEF0117 was tested in humans and was shown to reduce cannabis effects and cannabis self-administration in individuals with CUD in phase II studies (51) (Figure 2D).

Altogether, those findings suggest that, at this point, FAAH inhibitor may not be as effective as it was initially hoped. Nabiximols still have some important potential for CUD treatment. Among more recent drug in development, the CB_1_-SSi AEF0117 seems to have some potential for development and should be tested in treatment-seeking patients. Drugs such as neutral CB_1_ antagonist or NAMs may have some potential but would need to be tested in humans first. We can hope that one of those cannabinoid drugs may prove useful as medications for treating CUD in the future.

Other medications with potential utility include the anticonvulsant drugs gabapentin (NCT00395044) (150) and topiramate (NCT01110434) (151). Although other drugs have been tested in small-scale studies (e.g., opioid antagonists, n-acetylcysteine, oxytocin, and varenicline), it is unclear whether they have utility in treating CUD (140). The antipsychotic quietapine may be useful in specific population, but its antipsychotic profile may limit its broad utility (152).

## Conclusion

There have been tremendous advances in our understanding of CUD. Clearly, the determinants and risk factors are now better understood (1). We have also started to obtain insights into the neurobiological alterations associated with CUD. Advances in our preclinical models (41) and in the laboratory testing of cannabis self-administration (153) are allowing us to make faster progress on testing innovative treatment approaches for CUD (41). Harmonizing clinical trial outcome measures will be helpful for the field to compare results from clinical trials (154). The management of CUD relies on the usual approaches in addiction medicine (155), which so far are mostly psychosocial interventions. However, it seems likely that, in the coming years, pharmacological interventions will be validated and will complement psychosocial treatments delivered to patients with CUD (116). In addition, neuromodulations approaches (e.g., repeated transcranial magnetic stimulation) that have shown some promises for substance use disorder treatment (156, 157) are being explored as treatment modality for CUD (158) and may represent an alternative to pharmacotherapies in the future.

## Figures and Tables

**Table 2:**
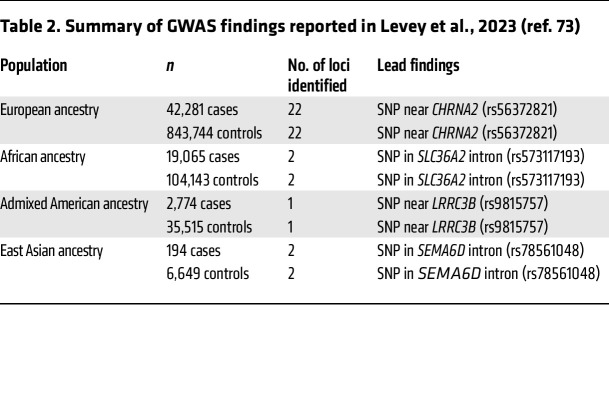
Summary of GWAS findings reported in Levey et al., 2023 (ref. 73)

**Table 1:**
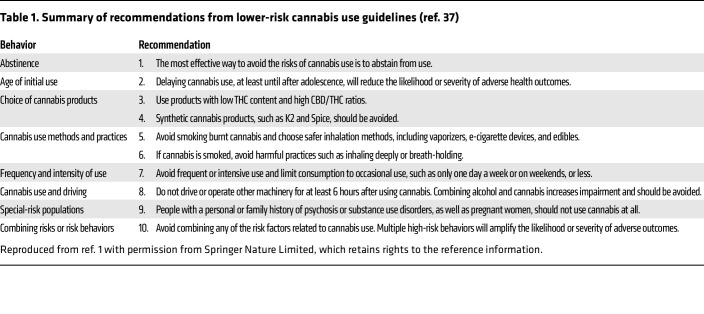
Summary of recommendations from lower-risk cannabis use guidelines (ref. 37)

## Version 1 10/15/2024

Electronic publication
